# Imaging assessment of glenohumeral dysplasia secondary to brachial plexus birth palsy*

**DOI:** 10.1590/0100-3984.2015.0039

**Published:** 2016

**Authors:** Francisco Abaete Chagas-Neto, Vitor Faeda Dalto, Michel Daoud Crema, Peter M. Waters, Everaldo Gregio-Junior, Nilton Mazzer, Marcello Henrique Nogueira-Barbosa

**Affiliations:** 1 Radiology Professor, Division of Radiology, Universidade de Fortaleza (Unifor) and Centro Universitário Christus, Fortaleza, CE, Brazil.; 2 PhD Student, Division of Radiology, Internal Medicine Department, Faculdade de Medicina de Ribeirão Preto da Universidade de São Paulo (FMRP-USP), Ribeirão Preto, SP, Brazil.; 3 MD, Radiologist, Radiology Department, Hôpital Saint-Antoine, Université Paris VI, Paris, France; Department of Radiology, Quantitative Imaging Center, Boston University School of Medicine, Boston, MA, USA; Department of Radiology and Tele-Imaging, Hospital do Coração (HCor), São Paulo, SP, Brazil.; 4 Orthopedic Surgeon-in-Chief, Brachial Plexus Program Director, Orthopedic Center, Boston Children's Hospital, Harvard Medical School, Boston, MA, USA.; 5 PhD, MD, Radiologist, União Médica Radiológica Catanduva (UMERC), Catanduva, SP, Brazil.; 6 Full Professor of Orthopedics, Department of Biomechanics, Medicine, and Rehabilitation of the Locomotor Apparatus, Faculdade de Medicina de Ribeirão Preto da Universidade de São Paulo (FMRP-USP), Ribeirão Preto, SP, Brazil.; 7 Associate Professor of Radiology, Division of Radiology, Internal Medicine Department, Faculdade de Medicina de Ribeirão Preto da Universidade de São Paulo (FMRP-USP), Ribeirão Preto, SP, Brazil.

**Keywords:** Birth injuries/complications, Joint diseases/diagnosis, Brachial plexus neuropathies/complications, Humeral head/abnormalities, Shoulder dislocation/diagnosis, Tomography

## Abstract

**Objective:**

To assess imaging parameters related to the morphology of the glenohumeral joint in children with unilateral brachial plexus birth palsy (BPBP), in comparison with those obtained for healthy shoulders.

**Materials and Methods:**

We conducted a retrospective search for cases of unilateral BPBP diagnosed at our facility. Only patients with a clinical diagnosis of unilateral BPBP were included, and the final study sample consisted of 10 consecutive patients who were assessed with cross-sectional imaging. The glenoid version, the translation of the humeral head, and the degrees of glenohumeral dysplasia were assessed.

**Results:**

The mean diameter of the affected humeral heads was 1.93 cm, compared with 2.33 cm for those of the normal limbs. In two cases, there was no significant posterior displacement of the humeral head, five cases showed posterior subluxation of the humeral head, and the remaining three cases showed total luxation of the humeral head. The mean glenoid version angle of the affected limbs (90-α) was -9.6º, versus +1.6º for the normal, contralateral limbs.

**Conclusion:**

The main deformities found in this study were BPBP-associated retroversion of the glenoid cavity, developmental delay of the humeral head, and posterior translation of the humeral head.

## Abstract (pt)

**Objetivo:**

Avaliar os parâmetros de imagem relacionados com a morfologia da articulação glenoumeral em crianças com paralisia obstétrica do plexo braquial (POPB) unilateral, comparando-os com os observados em ombros saudáveis.

**Materiais e Métodos:**

Foi realizada uma busca retrospectiva de casos de POPB unilateral diagnosticados em nossa instituição. Somente foram incluídos os pacientes com diagnóstico clínico de POPB unilateral, e a amostra final do estudo consistiu em 10 pacientes consecutivos avaliados por meio de imagens transversais. Foram avaliados a retroversão da cavidade glenoide, a translação da cabeça do úmero e o grau de displasia glenoumeral.

**Resultados:**

A média do diâmetro da cabeça do úmero foi 1,93 cm nos membros afetados e 2,33 cm nos membros normais. Em dois casos, não houve deslocamento posterior significativo da cabeça do úmero, cinco casos apresentaram subluxação posterior da cabeça do úmero, e os três casos restantes apresentaram luxação total da cabeça do úmero. A média do ângulo de retroversão glenoide dos membros afetados (90-α) foi -9,6º, ao passo que a dos membros contralaterais normais foi +1,6º.

**Conclusão:**

As principais deformidades encontradas neste estudo foram retroversão da cavidade glenoide relacionada com POPB, atraso no desenvolvimento da cabeça do úmero e translação posterior da cabeça do úmero.

## INTRODUCTION

Brachial plexus birth palsy (BPBP) most commonly affects the upper trunk nerve components of the brachial plexus (C5-T1) and may lead to severe dysfunction of the shoulder. BPBP represents a significant source of motor disability, causing morbidity due to the limited active functional range of motion and joint contractures of the upper limbs, with an incidence of approximately 1.5 per 1000 live births in the United States^(1)^.

Approximately 70% of obstetric injuries to the brachial plexus show spontaneous regression in the first months of life, although there is limited spontaneous recovery of motor function in the remaining 30%^(1)^. In cases of incomplete recovery during the first two to three years of life, changes in the glenoid cavity and in the humeral head can appear as early as the fifth month of life, due to contractures and muscle imbalance^(2)^. Initial deformities result from glenohumeral joint contractures and an imbalance in the muscles of the shoulder girdle, caused by paralysis of the external abductors and rotators, combined with relative hyperactivity of the internal adductors and internal rotators, which are partially spared from neurological involvement^(1-3)^.

The main bone alterations secondary to birth palsy of the brachial plexus are as follows: progressive retroversion of the glenoid cavity; thinning and loss of bone at the posterior border of the glenoid; posterior subluxation or luxation of the humeral head; hypoplasia of the scapula; flattening or absence of the glenoid cavity; inferior deviation of the coracoid process; conic deformation of the acromion; deformity and hypoplasia of the humeral head; delayed bone development in the proximal humerus; and shortening of the clavicle in comparison with that of the contralateral side.

Because of the risk of rapid progression of BPBP, early diagnosis and therapeutic interventions are critical. Several studies have evaluated different ways to quantify the degrees of glenoid anteversion and retroversion, as well as to measure the different degrees of translation of the humeral head^(4-7)^.

The aim of our study was to assess imaging parameters related to the morphology of the glenohumeral joint in children with unilateral BPBP, comparing these parameters between pathological and healthy shoulders.

## MATERIALS AND METHODS

### Subject selection and clinical data

This study was approved by the local institutional review board, which conceded an exemption from the requirement to obtain informed consent. We conducted a retrospective search for cases of unilateral BPBP diagnosed at our service. The identification of cases was based on searches of reports from computed tomography (CT) and magnetic resonance imaging (MRI) studies of the shoulders of children, conducted between January 1, 2005, and December 31, 2010, in radiology information systems. We used the following search terms: "glenoid retroversion"; "glenoid dysplasia"; "posterior subluxation or luxation of the humeral head"; "brachial plexus lesion"; and "hypoplasia of the humeral head". Reports and images were reviewed and correlated with additional imaging reports, as well as with the clinical and surgical history. Only patients with a clinical diagnosis of unilateral BPBP were included. Exclusion criteria were being > 12 years of age; showing clinical or imaging signs of bilateral neurological injury; having undergone MRI or CT in which the results were suboptimal (because of motion artifacts, failure to complete the examination, or suboptimal patient positioning); showing signs of infectious or inflammatory arthritis; and showing evidence of prior shoulder surgery. The final study sample consisted of 10 consecutive patients with unilateral BPBP who were assessed with cross-sectional imaging via CT or MRI.

### Imaging studies

All examinations were performed according to our institutional routine protocol. Patients were placed in the supine position, with both shoulders supported on the table and the arms relaxed in the neutral position.

All MRI scans were obtained in the same 1.5 T MRI scanner (Philips Achieva 1.5 T MRI System; Philips Medical Systems, Best, the Netherlands) with a phased array coil. For each shoulder, coronal and sagittal T1-weighted images were obtained (3.0 mm slices; 0.5 mm gap), as were axial, sagittal, and coronal T2-weighted images. Field of view was adjusted to the size of the child, and the matrix size was 256 × 256.

CT scans were obtained in either a 16-channel multislice scanner (Philips Brilliance CT Big Bore System; Philips Medical Systems) or a single channel helical CT scanner (Somatom Emotion; Siemens Healthcare, Erlangen, Germany). Axial images of the shoulders (1.25 mm slices; 0.625 mm gap) were obtained and then reformatted in the axial, sagittal, and coronal planes (2.0 mm slices). Field of view, kV, and mA were adjusted to the size of the child.

### Reading technique and quantitative parameters

Axial plane images from the MRI and CT examinations were evaluated on a picture archiving and communication system, in consensus, by two fellowship-trained musculoskeletal radiologists with 5 and 15 years of experience, respectively.

**Figure 1:**
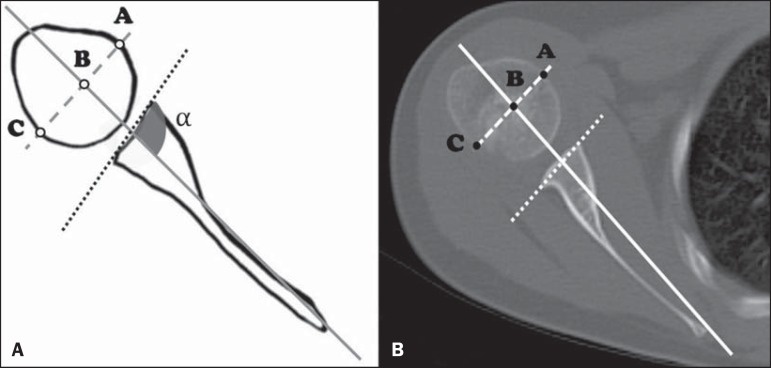
**A:** Schematic drawing of glenoid version (90-α) and humeral head translation measurement methods. The percentage of the humeral head anterior to the scapular line (PHHA) was measured according to the formula *PHHA = AB/AC* × 100. **B:** Axial computed tomography scan of the glenohumeral joint, corresponding to an illustrative case.

The glenoid version was assessed using the method described by Friedman et al.^(6)^: the angle formed by the line drawn at the anterior and posterior aspects of the glenoid margins and the line between the medial margin of the scapula and the midpoint of the glenoid is measured, then 90º is subtracted from the resulting angle (Figure 1). Both lines were drawn in the same image slice. Positive values were interpreted as glenoid cavity anteversion, and negative values were interpreted as glenoid cavity retroversion.

The measurement of the translation of the humeral head, represented by the percentage of the humeral head anterior to the scapular line (PHHA), was performed as described by Waters et al.^(8)^: we measured the distance between the margins of the humeral head by drawing a line perpendicular to the line of the scapula body (as in the Friedman method) at the midpoint of the humeral head, thus obtaining the ratio between the measurements (*PHHA = AB/AC* × 100) (Figure 1). Humeral head displacement was considered insignificant when the PHHA values were between 40% and 50%; posterior subluxation was defined as a PHHA between 0% and 35%; and the entire humeral head being posterior to the scapular line was classified as total luxation. The greatest axial diameter of the affected and contralateral humeral heads was also measured by the method shown in Figure 1 (AC distance).

The axial slice level used for the measurements was defined separately for the affected and normal contralateral sides as the first image below the coracoid process that best showed the reference anatomical structures (glenoid margins, scapula margins, and humeral head). After the measurements, the degrees of glenohumeral dysplasia were classified according to the criteria established by Waters et al.^(8)^, as shown in Figure 1.

### Statistical analysis

Statistical analyses were performed with KyPlot 2.0^TM^ software, to compare parameters assessed between BPBP-affected limbs and unaffected, contralateral limbs. Statistical significance was defined as a *p* < 0.05. Unaffected, contralateral limbs were used as internal controls for this study.

## RESULTS

Six males and four females, with a mean age of four years and nine months (range, 2-12 years), were included in this study. In six cases, the left shoulder was affected. CT and MRI were used in the evaluation of eight cases and four cases, respectively, both having been used in two cases.

**Figure 2:**
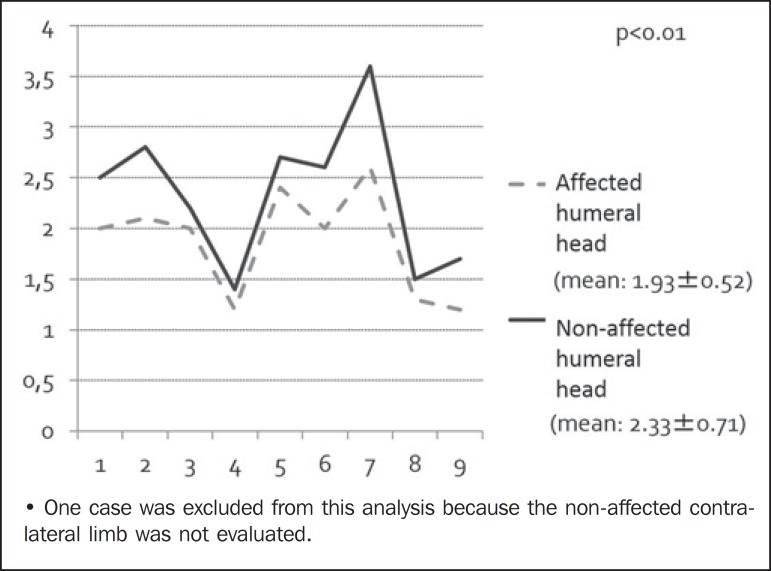
Sample distribution regarding the diameter of the humeral head (cm) on the affected side and unaffected (contralateral) side.

The mean diameter of the affected humeral head was 1.93 cm, with a standard deviation of 0.52 cm. The contralateral humeral head measured an average of 2.33 cm, with a standard deviation of 0.71 cm. In all patients, there was marked asymmetry in the humeral head diameters and the difference was statistically significant (*p* < 0.01). This indicates a developmental delay in the humeral head on the affected side (Figure 2).

Two cases showed no significant posterior displacement of the humeral head, five cases showed posterior subluxation of the humeral head, and the three remaining cases showed total luxation of the humeral head.

**Figure 3:**
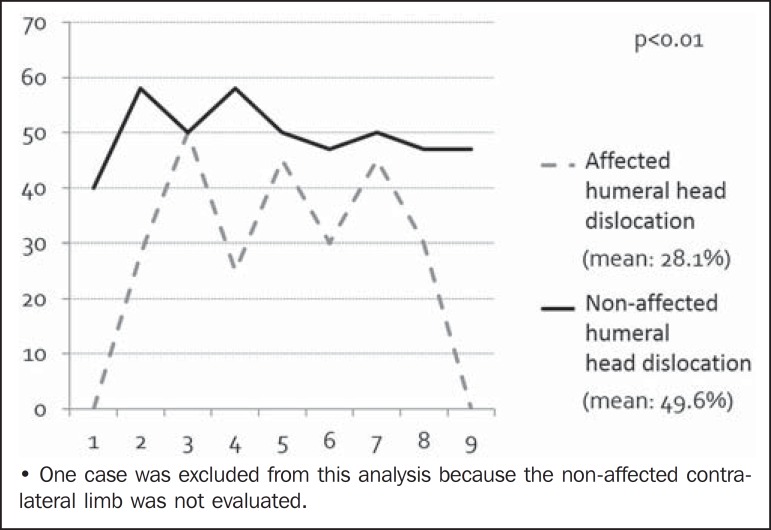
Sample distribution regarding the percentage of the humeral head anterior to the scapular line. A reference range for normality would be 40-50%.

The mean PHHA for the affected shoulder was 24%, with a standard deviation of 18%, compared with 48% with standard deviation of 6%, for the contralateral shoulder. As can be seen in Figure 3, the posterior translation of the humeral head was significant greater on the affected side (*p* < 0.05).

**Figure 4:**
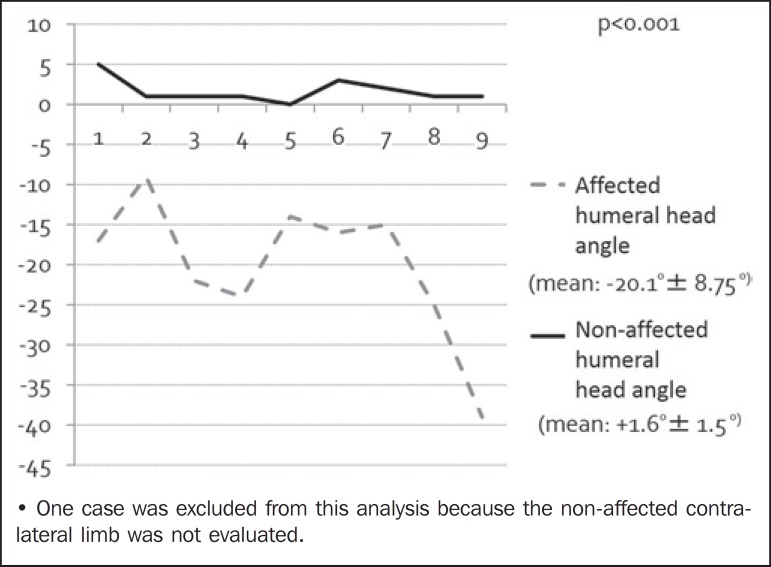
Glenoid version distribution on the affected side and unaffected (contralateral) side.

**Figure 5:**
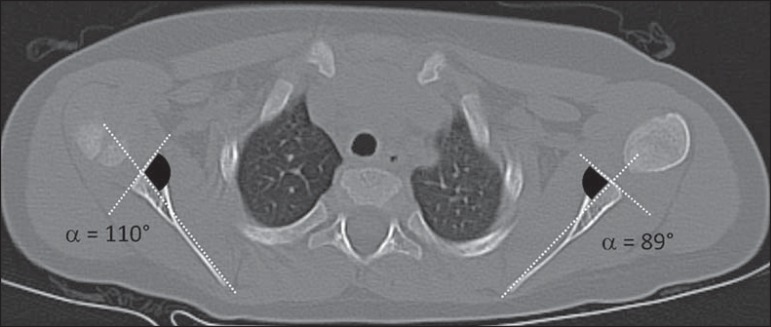
Illustrative example of glenohumeral dysplasia shown in an axial section computed tomography scan. The right glenohumeral joint was dysplastic and the left side was unaffected. The angles of retroversion were measured as described by Friedman et al.^(6)^. Right side: α = 110º; version angle: 90-110º = -20º, interpreted as glenoid cavity retroversion. Left side: α = 89º; version angle: 90-89º = +1º, interpreted as glenoid cavity anteversion (normal contralateral).

The average glenoid version of the affected limbs (90-α) was -19.6º (95% CI: -11.1º to -28.0º) versus +1.6º (95% CI: 1.0º to 3.1º) in the normal contralateral limbs. In all patients, the affected side presented retroversion of the glenoid cavity, varying between -9º and -39º, and was significantly different from the unaffected side (*p* < 0.05), as depicted in Figures 4 and 5.

According to the classification system devised by Waters et al.^(8)^, detailed in Table 1, our sample presented two cases with mild deformity (Waters type II), three cases with moderate deformity (Waters type III), two cases with severe deformity (Waters type IV), and three cases of total posterior displacement of the humeral head (Waters type VI). In our sample, there were no cases classified as Waters types I, V, or VII.

**Table 1:** Deformity of the glenohumeral joint according to the criteria established by Waters et al.^(8)^.

Classification	Description
Type I (normal glenoid)	Less than 5º difference in retroversion between affected and unaffected glenoid
Type II (mild deformity)	More than 5º difference in retroversion between affected and unaffected glenoid
Type III (moderate deformity)	Posterior subluxation of humeral head. Less than 35% of head is anterior to the scapular line
Type IV (severe deformity)	Presence of false glenoid
Type V	Severe humeral head and glenoid flattening with progressive or complete humeral head posterior dislocation
Type VI	Posterior humeral head dislocation in infancy
Type VII	Growth arrest of proximal aspect of humerus

## DISCUSSION

Glenohumeral dysplasia secondary to BPBP is an uncommon cause of severe limb dysfunction in young patients. Here, we have retrospectively reviewed 10 cases of BPBP. The main bone and articular alterations identified in those patients were posterior translation/subluxation of the humeral head, retroversion of the glenoid cavity, and developmental delay in the humeral head. The bone and anatomic alterations were statistically significant when compared against the contralateral unaffected limb (internal control).

The prevalence of bone deformities in BPBP varies across studies depending on the methodology and the evaluation criteria used. In a population-based study conducted by Sjöberg et al. in Sweden^(9)^, approximately 25% of births with brachial plexus palsy were found to present persistent abnormalities. In a prospective cohort assessing 94 children with brachial plexus palsy, the authors found abnormalities on cross-sectional imaging in 38% of the cases, and 26 (62%) of the 42 patients who underwent tomography for surgical planning presented evidence of posterior subluxation of the humeral head^(10)^.

In the present study, approximately 80% of the patients presented posterior subluxation or total luxation of the humeral head (Waters types III, IV, and VI). Only two cases (20%) presented mild deformities (Waters type II). The high frequency of advanced cases in our sample, compared with what has been reported in the literature^(9,10)^, may be related to increased referral of highly complex cases, underdiagnosis of mild cases, or late diagnosis with unfavorable development.

Recent studies have emphasized the importance of imaging methods (ultrasound, CT, and MRI) in the early evaluation and classification of patients with brachial obstetrical plexus palsy^(11-16)^. Multiple methods for measurement and classification of the glenoid dysplasia and its associated findings have been described in the literature. The methods used in this study were previously described by Friedman et al.^(6)^ and Waters et al.^(8)^, which are notable for their accuracy and reproducibility^(3,6,8)^.

There is as yet no consensus in the literature regarding the role of different imaging methods in the diagnosis and monitoring of BPBP. Imaging assessment in the first three months after birth is rare because most cases present spontaneous clinical remission. In infants between three and eighteen months of age, ultrasound may be used as a screening test in those who did not present satisfactory progress or who present with clinical worsening^(16)^.

**Figure 6:**
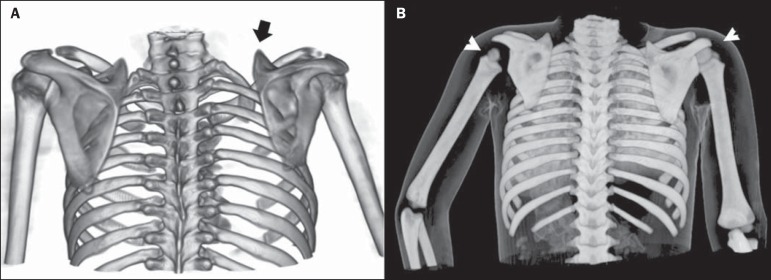
Examples of three-dimensional volume rendering of computed tomography images acquired from two different patients. **A:** Three-dimensional reconstruction, posterior view. Female patient, 12 years old, history of high obstetric traumatic brachial plexus injury on the right. Note the elevation of the right scapula (arrow) and reduction of right scapula size. **B:** Posterior view. Volume rendering with skin referential of a 4-year-old male patient with obstetric brachial plexus injury on the left side. Note the elevation of the left scapula, reduced size of the humeral head (arrowheads), and positioning of the left limb (maintained in abduction and internal rotation).

The importance of MRI in patients over 18 months of age should be highlighted, because it allows for morphological assessment, location of the humeral head epiphyseal cartilage, and aids in the mapping of muscular structures for surgical planning. CT can be used as a complementary, or even standalone, technique for detailed evaluation of bone structures (Figure 6).

Proper evaluation of sectional imaging parameters, as described in this study, is important for correct classification of dysplasia severity and has a direct effect on treatment choice and post-surgical prognosis for patients^(17-22)^. Patients with minimal (Waters type I) glenohumeral dysplasiawill benefit from latissimus dorsi and teres major tendon transference to the rotator cuff. Patients with mild or moderate (Waters type II or III) glenohumeral dysplasia, with reducible subluxation or luxation, will be better served by treatment with open or arthroscopic reduction of the glenohumeral joint, together with latissimus dorsi and teres major tendon transference to the rotator cuff, as well as lengthening of the pectoralis major tendon, subscapularis tendon, or both. Finally, older patients with severe (Waters type IV or higher) glenohumeral deformity, irreducible glenohumeral luxation, or established arthrosis are candidates for humeral osteotomy^(17-22)^. Recently, a combination of ante-version glenoid osteotomy, so-called glenoplasty, and tendon transfer has come to be used as a therapeutic option in younger patients with moderate to severe glenohumeral dysplasia^(23)^.

The main limitations of this study are the retrospective design and the small sample size. In addition, images were not acquired using a standardized protocol and evaluations were performed on a mix of CT and MRI images. Furthermore, readings were performed in tandem, and it was therefore not possible to evaluate interobserver agreement. Despite these limiting factors, the findings presented in this study demonstrate a useful and easily adoptable method for the evaluation of imaging studies in cases of BPBP.

## CONCLUSION

The main deformities found in the affected limbs evaluated in this study were BPBP-associated retroversion of the glenoid cavity, delayed development of the humeral head, and posterior translation of the humeral head. It is essential that the radiologists become familiar with this condition and its presentation, as well as with the diagnostic methods available for its detection and classification. Such assessments may have significant therapeutic and prognostic implications in patients affected by BPBP.
